# Escores para o Diagnóstico da Etiologia Maligna do Derrame Pericárdico: Uma Valiosa Ajuda Inicial na Investigação

**DOI:** 10.36660/abc.20230762

**Published:** 2024-02-16

**Authors:** Wolney de Andrade Martins

**Affiliations:** 1 Universidade Federal Fluminense Niterói RJ Brasil Curso de Pós-Graduação em Ciências Cardiovasculares da Universidade Federal Fluminense, Niterói, RJ - Brasil; 2 Universidade do Coração Instituto Nacional de Câncer Instituto Nacional de Cardiologia Rio de Janeiro RJ Brasil Curso de Pós-Graduação em Cardio-Oncologia, Universidade do Coração, Sociedade Brasileira de Cardiologia – Instituto Nacional de Cardiologia – Instituto Nacional de Câncer, Rio de Janeiro, RJ – Brasil

**Keywords:** Derrame Pericárdico, Tamponamento Cardíaco, Mortalidade, Neoplasias, Prognóstico, Metastase/complicações, Síndrome Coronariana Aguda

Doenças no pericárdio são prevalentes em pacientes com câncer (CA). A presença de derrame pericárdico (DP) nestes pacientes seguramente piora o prognóstico. A possibilidade do tamponamento cardíaco acrescenta risco de morte.

O artigo “*Testes de Triagem Prevendo Metástase de Câncer na Etiologia do Derrame Pericárdico: HALP Score e PNI”*^1^ publicado nos ABC Cardiol traz à discussão o dilema clínico da etiologia neoplásica do DP. A facilidade de acesso aos métodos de imagem tornou frequente o diagnóstico do DP nos pacientes com CA e tornou tentadora a busca etiológica. Não raramente nos deparamos com DP de etiologia inconclusiva após exaustiva investigação. Por outro lado, o temor de deixar de fazer o diagnóstico de um CA oculto é preocupante. Sabemos que o DP no paciente com CA pode ser secundário ao implante metastático, à invasão direta do tumor, à obstrução linfática, à pericardite causada pela quimioterapia, imunoterapia ou radioterapia e à pericardite de causas infecciosas comuns em imunodeprimidos.^2^ Ademais não se podem descartar outras causas tais como doença renal, hipotireoidismo, insuficiência cardíaca, doenças autoimunes, correlatas ao CA ou não.^3^

Alguns escores têm sido propostos, avaliados e validados para predizerem prognóstico em pacientes com diversos tumores sólidos ou hematológicos. A maioria se baseia na intensa e mantida inflamação sistêmica e desnutrição ou catabolismo presentes nos pacientes com CA. Uma meta-análise recente evidenciou que o HALP escore foi capaz de predizer sobrevida em pacientes com tumores sólidos.^4,5^ Os escores estudados neste artigo dos ABC também têm sido aplicados para aferir prognóstico em síndromes cardiovasculares como a síndrome coronariana aguda^6^ e pacientes com acidente vascular cerebral.^7^

Neste artigo,^1^ 283 pacientes com DP foram avaliados retrospectivamente em casuística acumulada em longos 16 anos de observação. Dentre os pacientes com DP, 30% tiveram CA como etiologia, com predomínio do CA de pulmão e consequentemente o gênero masculino.

A ideia central do artigo,^1^ com importante aplicabilidade clínica, é predizer a etiologia neoplásica a partir de escores de fácil obtenção derivados de variáveis acessíveis. Estas variáveis são secundárias ao estado inflamatório sistêmico e ao hipercatabolismo presente nos pacientes com CA. Foram estudados três escores que combinam a contagem de linfócitos, plaquetas, albumina, hemácias, neutrófilos, geralmente diminuídos no paciente com CA e a dosagem elevada da PCR. Duas formulações tiveram melhor desempenho em termos de sensibilidade e especificidade para predizerem a etiologia neoplásica. Elas foram o HALP e o PNI. Deve-se lembrar que outros biomarcadores podem auxiliar no diagnóstico diferencial da etiologia do DP. A troponina se eleva no comprometimento miopericárdico de qualquer etiologia. A velocidade de hemossedimentação se eleva nas etiologias inflamatórias. Marcadores de doenças reumatológicas podem ser úteis. Pelo artigo publicado,^1^ conclui-se que os escores HALP e PNI funcionariam como indicativos da necessidade de investigação mais dedicada para um CA subjacente, especialmente o CA de pulmão. Frente a suspeição de DP por metástase, os métodos de imagem se tornam mandatórios.

Os resultados ora apresentados nos permitem especular se tais escores também estariam alterados e funcionariam como preditores de CA em outras alterações cardiovasculares secundárias ao CA, tais como na doença tromboembólica, nas massas cardíacas, no diagnóstico diferencial da endocardite infecciosa com trombo. Obviamente necessitamos de outros estudos direcionados.

Ganhamos aqui neste artigo dos ABC uma possibilidade acessível e não invasiva de aumentar a probabilidade do diagnóstico da etiologia neoplásica nos derrames pericárdicos dos pacientes com CA.
