# Supplementary material for: Escores para o Diagnóstico da Etiologia Maligna do Derrame Pericárdico: Uma Valiosa Ajuda Inicial na Investigação
Source: Arq Bras Cardiol. 2024 Feb 16;121(1):e20230762. [Article in Portuguese] doi: 10.36660/abc.20230762 (PMC11081061; doi:10.36660/abc.20230762)
Supplement: Translation

# Translation: Scores for Diagnosing the Malignant Etiology of Pericardial Effusion: A Valuable Initial Aid in the Investigation

**Authors:** Wolney de Andrade Martins

**Affiliations:** 1 Niterói RJ Brazil Curso de Pós-Graduação em Ciências Cardiovasculares da Universidade Federal Fluminense, Niterói, RJ – Brazil; 2 Rio de Janeiro RJ Brazil Curso de Pós-Graduação em Cardio-Oncologia, Universidade do Coração, Sociedade Brasileira de Cardiologia – Instituto Nacional de Cardiologia – Instituto Nacional de Câncer, Rio de Janeiro, RJ – Brazil

**Keywords:** Pericardial Effusion, Cardiac Tamponade, Mortality, Neoplasms, Prognosis, Acute Coronary Syndrome

Pericardial diseases are prevalent in cancer (CA) patients. The presence of pericardial effusion (PE) in these patients certainly worsens the prognosis. The possibility of cardiac tamponade increases the risk of death.

The article ‘*Screening Tests Predicting Cancer Metastasis in the Etiology of Pericardial Effusion: HALP Score and PNI*’^1^ published in ABC, brings to discussion the clinical dilemma of the neoplastic etiology of PE. The ease of access to imaging methods has made the diagnosis of PE common in patients with CA and made the etiological search tempting. It is not uncommon to come across PE with an inconclusive etiology after an exhaustive investigation. On the other hand, the fear of failing to diagnose a hidden CA is worrying. We know that PE in patients with CA can be secondary to metastatic implantation, direct invasion of the tumor, lymphatic obstruction, pericarditis caused by chemotherapy, immunotherapy, or radiotherapy, and pericarditis from infectious causes common in immunocompromised patients.^2^ Furthermore, other causes cannot be ruled out, such as kidney disease, hypothyroidism, heart failure, and autoimmune diseases, whether related to CA or not.^3^

Some scores have been proposed, evaluated, and validated to predict prognosis in patients with various solid or hematological tumors. Most are based on the intense and sustained systemic inflammation and malnutrition or catabolism present in patients with CA. A recent meta-analysis showed that the HALP score was able to predict survival in patients with solid tumors.^4,5^ The scores studied in this article published in ABC have also been applied to assess prognosis in cardiovascular syndromes such as acute coronary syndrome^6^ and patients with stroke.^7^

In this article,^1^ 283 patients with PE were retrospectively evaluated in a case series accumulated over 16 years of observation. Among patients with PE, 30% had CA as the etiology, with a predominance of lung CA and, consequently, the male gender.

The central idea of the article,^1^ with important clinical applicability, is to predict neoplastic etiology based on easily obtained scores derived from accessible variables. These variables are secondary to the systemic inflammatory state and hypercatabolism present in patients with CA. Three scores were studied that combined the count of lymphocytes, platelets, albumin, red blood cells, and neutrophils, generally reduced in patients with CA and high CRP dosage. Two formulations performed better in terms of sensitivity and specificity for predicting neoplastic etiology. They were HALP and PNI. It should be remembered that other biomarkers can help in the differential diagnosis of PE etiology. Troponin rises in myopericardial involvement of any etiology. The erythrocyte sedimentation rate increases in inflammatory etiologies. Markers of rheumatological diseases may be useful. From the published article,^1^ it is concluded that the HALP and PNI scores would act as indicators of the need for more dedicated investigation into an underlying CA, especially lung CA. When PE is initially suspected to be due to metastasis, imaging methods become mandatory.

The results presented here allow us to speculate whether such scores would also be altered and would function as predictors of CA in other cardiovascular changes secondary to CA, such as thromboembolic disease, cardiac masses, and the differential diagnosis of infective endocarditis with thrombus. Obviously, we need other targeted studies.

Here in this ABC paper, we gain an accessible and non-invasive possibility of increasing the probability of diagnosing neoplastic etiology in pericardial effusions in cancer patients.
